# Genetic Impact on Bone Modulation—A Review Bridging Bioscience to Genetic Engineering

**DOI:** 10.1055/s-0041-1725069

**Published:** 2021-03-10

**Authors:** Anulekha Avinash CK, Harshini Tholupunuri, M. Ramu Reddy, Mamatha Muralidhar, Dilip Jayyarapu, Sangeeta Nair

**Affiliations:** 1 Kamineni Institute of Dental Sciences, Narketpally, Telangana, India; 2 Department of Prosthodontics, Kamineni Institute of Dental Sciences, Narketpally, Telangana, India; 3 Department of Prosthodontics Anoor Dental College, Muvattupuzha, Kerala, India

**Keywords:** Keywords, genetic mutations syndromes bone resorption

## Abstract

Genes control approximately 60% to 75% of the variance of peak bone mass/density and a much smaller amount of variance in rate of loss.

Bone mass increases during growth to a peak value and soon after begins to decline. Most of the genetic effect is exerted during growth and so influences peak bone mass; whether there is an additional genetic effect on the rate of bone loss is less clear. So, this article aims to place emphasis on various oral and systemic conditions that are manifested due to altered gene function. Genetic polymorphisms and mutations are simple, although the consequences of the mechanism are complex. The syndromic manifestation due to changes at genetic level will greatly affect the bone quality, which will ultimately affect any treatment prognosis. Hence, a better understanding of molecular mechanisms of bone remodeling helps to identify pathogenic causes of bone, skeletal diseases, and leads to the development of targeted therapies for these diseases. This review highlights notions on the connecting link between science and genetics as well as various oral scenarios where gene could bring about changes, resulting in deformities. There is an intense research awaited in the future which could intervene with the causes that bring about genetic modulations, so as to decrease the mortality rate of humans.

## Introduction

Bone remodeling is a physiological process; if there exists an imbalance in the metabolic activities of the cells, the process turns to a pathological condition. Bone formation and resorption is a common process throughout the age, unless and until the process is disturbed by an etiology like hormonal disturbances, developmental anomalies, genetic variations, and so on. 1 2 3 More often, ridge resorption is a common problem encountered with the elderly age group. This scenario is extremely challenging with prosthodontic view, as the available basal foundation is compromised to retain and support the prosthesis. Surgical intervention also becomes challenging due to fear of violating the vital structures. Relating the resorption to an oral scenario, one could describe the residual ridge as the shape of the clinical alveolar ridge after healing of bone and soft tissues posttooth extractions. A cascade of inflammatory reactions is activated immediately after tooth extraction, and the extraction socket is temporarily closed by the blood clotting. 4 It is documented that residual ridge resorption is multifactorial by origin, which involves anatomic, metabolic, and mechanical factors. 5 However, the resorption to get accelerated requires the involvement of systemic conditions of health. With progression in age, especially females of menopausal age group undergo a lot of physiological changes, starting from the genetic level. 6 7 8

The influence of the genes varies, bringing about changes at various levels of health. The ill effects of genetic changes, affecting various systems of the human body, is deleterious in its own way. However, our focus is particularly on the genetic changes influencing the skeletal system, especially the alveolar ridges. The quality and quantity of residual ridge is extremely significant, so as to sustain a healthy abutment around it as well as support the artificial prosthesis in areas where there is a need to substitute the missing teeth. Hence, the greater the quality of bone, better would be the prognosis of the dental treatment. 9

There is a tremendous literature support which strongly proves that a genetic factor links an association between periodontitis, osteoporosis, and few syndromes that cause bone modulation. 10 11 12 However, in all of the above-mentioned studies, the role of gene is vital. There is a need to study about the genetic factors, so as to explore the probabilities that could be considered in intervening with progression of the disease. The role of gene that is involved in bringing about the resorptive changes is complex and extremely interesting. Although there is documentation regarding the influence of various genes involved in creating various pathological changes in the human body, a considerable amount of research still needs to be conducted with regard to genetic factors involved which create and lead to progress bony changes.

Hence, an effort is made to gather the information regarding various types of genes involved in bone modulation of various types of compromised health conditions, affecting the harmony of the stomatognathic system. The article describes various health conditions where genes place emphasis on creating a compromise in health and devastating the existing condition. There is a possibility in future to intervene with the causes that bring about genetic changes, so as to decrease the mortality rate of humans. The advancement in the genetic engineering is likely to bring about a huge leap in the field of molecular genetics.

Virtually every single disease in human beings is directly or indirectly affected by genetic variation, conferring susceptibility, resistance, or interaction with environmental factors. Single nucleotide polymorphism (SNP) is the most common type of genetic variation, in which there is an alternate base occurring at a frequency greater than 1% in the human population. 13

The correlation between SNPs in vascular endothelial growth factor and residual ridge resorption (RRR) was studied by Song and Lee among 120 Korean subjects with the edentulous mandible, observing a conspicuous relation between rs1570360 and haplotype A-C-C with RRR. The investigators affirm it as an unprecedented genetic marker for identifying people susceptible to severe RRR after dental extraction. 14 Similarly, AlSheikh et al in a study not only showed an association between SNPs in IL10 and NOD2 genes but also revealed that the genotypes of the different SNPs affect health and bone resorption. Hence, identifying SNPs in genes which are vulnerable would not only allow us to have a better understanding of disease processes and their underlying mechanisms but also provide scope for more efficient treatment options against RRR. 15

In the quest to unveil the role of genetic factors in the development of RRR, there has been few studies which investigated SNPs in different genes, including cytokine and growth factor genes, genes of matrix metalloproteinases (MMPs), fibroblast growth factor receptor (FGFR1), and hypoxia-inducible factor-1 (HIF-1α) and have showed marked associations between some polymorphisms and RRR. 16 17 18 Sundar et al reported a positive correlation in terms of genetic association between SNPs of matrix metalloproteinase-1 (MMP-1) gene promoter and RRR of edentulous mandible. 14

## Pathogenesis Associated with Various Conditions

### After Tooth Extraction

Normally bone undergoes continuous process of remodeling all the time through bone resorption and bone deposition. As mentioned earlier, the moment a tooth is extracted, there is injury to the blood vessels, followed by blocking of blood which, in turn, results in reduction of partial pressure of oxygen in the surrounding area. Now, as the tissues are in the state of hypoxia, this is when genetic factors come into the scene. HIF-1 is a transcriptional complex that plays an important role in cellular and systemic oxygen homeostasis. HIF-1 consists of α and β subunits. The α subunit, which determines HIF-1 activity, is regulated by oxygen tension. Since HIF-1 α gene is critical for oral-wound healing after extraction of teeth, and since it has a high genetic diversity, common SNPs in HIF-1α could play a role in the appearance of severely resorbed mandibular ridges. 19 20 21

At the macroscopic level, during early healing period of dental extraction wounds, the gingival margins contract toward the center of the extraction socket and there is reestablishment of epithelial integrity. This newly regenerated epithelium becomes a central part of the edentulous oral mucosa. It is relatively thick with elongated rete pegs suggestive of acanthosis. But in stark contrast, thickness of the connective tissue of the established residual ridge is found decreased in both denture wearers and nondenture wearers, whereas the collagen density is increased. 22 23

All the above-mentioned findings highlight the fact that there is constant remodeling of the oral mucosa and contraction of connective tissue, which may result in a thin oral mucosa, a characteristic of atrophic edentulous residual ridge. 24

### Periodontitis

Another scenario where we see trademark bone loss is progressing periodontitis, and failure to prevent it poses a big challenge in providing effective treatment. Aggressive periodontitis which is also called “juvenile periodontitis” is considered to be prevalent in children and adolescents during circumpubertal period. It is characterized by rapid loss of connective tissue attachment and alveolar bone with familial aggregation. It is caused by both pathogenic microflora and abnormality in host defense mechanisms. Aggressive periodontitis can be localized or generalized. 25 Localized aggressive periodontitis (LAgP) occurs in children and adolescents without clinical evidence of systemic disease and is characterized by the severe loss of alveolar bone around permanent teeth. 26 Linkage studies of the Brandywine population (a segregated group of people in Maryland that represents a relatively closed gene pool) have found a gene conferring increased risk for LAgP on chromosome 4. 27

In periodontal disease, the cellular inflammatory infiltrate of T cells, B cells, macrophages, and neutrophils within gingival connective tissue is increased which, in turn, leads to increased secretion of inflammatory mediators. 28 These inflammatory cells also interact with stromal cells such as osteoblasts, periodontal ligament, and gingival fibroblasts. Bone resorption is primarily regulated by RANK signaling pathway, which plays a central role in osteoclast differentiate and function. Receptor activator of nuclear factor-kB ligand (RANKL) produced by lymphocytes do not play any role during normal conditions. But in an inflammatory pathological state such as periodontitis, it plays a major role in inflammatory bone resorption, which is mediated by activated T lymphocytes and B lymphocytes. These activated lymphocytes are found in large numbers in inflamed periodontal tissue. 29 30

### Osteoporosis

Osteoporosis is a common disease characterized by low-bone mass and defects in the microarchitecture of bone tissue, which impairs bone strength and leads to an increased risk of fragility fractures. Table 1 shows several rare diseases that have been identified where profound effects on bone mass, bone fragility, and bone turnover occur as a result of mutations in single genes.

**Table 1:** Influence of genetic mutation in various diseases

Disease	Gene	Defect in function
High bone mass syndrome	LRP5a	Increases bone formation and inhibits bone resorption by regulating OPG production by osteoblast
Aromatase deficiency	CYP17	Osteoporosis
Estrogen receptor deficiency	ESR1	Osteoporosis, tall stature
Osteoporosis-pseudoglioma syndrome	LRP5 gene	Low-bone mass and increased bone fragility

Abbreviation: OPG, osteoprotegerin.

## Loci and Genes with Significant Evidence for Association with Bone Mineral Density (BMD)

### ESR1

Estrogen, by interacting with its receptors in bone and other tissues, plays an important role in regulating skeletal growth and maintaining bone mass. The estrogen receptor type 1 gene (ESR1) is therefore a strong candidate for the genetic regulation of bone mass.

The association between ESR1 alleles and osteoporosis was confirmed by the deCODE GWAS, which showed a significant association with BMD and fracture. 31 32

The osteoporosis-pseudoglioma syndrome is a rare recessive disorder characterized by low-bone mass and increased bone fragility that has been found to be caused by inactivating mutations in the LRP5 gene. Many different gene mutations like TCIRG1, CLCN7, OSTM1, and NEMO have been identified in osteoclast-rich osteopetrosis, all of which impair the ability of osteoclasts to resorb bone. 33

### SOST

The SOST gene on chromosome 17q21 encodes sclerostin, a protein that is produced almost exclusively by osteocytes and which inhibits bone formation, probably by preventing members of the Wnt family binding to the LRP5 receptor. 34

### TNFRSF11B

The TNFRSF11B gene on chromosome 8 encodes osteoprotegerin (OPG), which is an endogenously produced inhibitor of bone resorption. 35 OPG plays a critical role in bone metabolism and has been the subject of several candidate gene association studies. 36

## Other Candidate Genes for Susceptibility to Osteoporosis

### COL1A1

Type I collagen is the major protein of bone and is also a heterotrimer consisting of α 1 and α 2 protein chains that are encoded by the COL1A1 and COL1A2 genes, respectively.

Polymorphisms of the COL1A1 gene have been studied extensively in relation to BMD and osteoporotic fracture.

The “T” allele of this polymorphism has been associated with BMD and/or osteoporotic fractures in several studies, 37 38 but negative results have also been reported. 39 40

Nonetheless, a retrospective meta-analysis of published studies showed that the COL1A1 Sp1 polymorphism was significantly associated with osteoporotic fractures 41 and bone density, 42 43 with evidence of an allele dose effect.

### TGFB1

TGF1 encoded by the TGFB1 gene is thought to act as F, a coupling factor between bone resorption and bone formation. This gene is been associated with low BMD, increased bone turnover, and osteoporotic fracture in one study from Denmark. 44

### VDR

The active metabolites of vitamin D play an important role in regulating bone cell function and maintaining serum calcium homeostasis by binding to the vitamin D receptor (VDR), which regulates expression of various response genes. The VDR gene has been extensively studied as a potential candidate for regulating genetic susceptibility to osteoporosis. Large number of association studies between VDR alleles and BMD and/or fracture were subsequently performed, but the results were conflicting, probably because none of the studies was adequately powered. 45

## Syndromes Associated Due to Genetic Impairment

The genetic impact on syndromes is tremendous, and unfortunately it leads to not only suffering lasting for a lifetime but also continues for many generations. Genes not only mediates inflammatory reactions but they are also responsible for various syndromes.

### Papillon–Lefevre Syndrome

Papillon–Lefevre syndrome is one such syndrome, characterized by different dermatological manifestations, with periodontitis being presented as systemic manifestation. It is an autosomal recessive condition that impairs the patient socially and psychologically and also his/her aesthetic well-being at a very young age, due to partial or complete edentulism. It is thought to be as a secondary result to the mutation of the cathepsin C gene.

Various studies have shown that the immune-related cells like the polymorphonuclear leucocytes and the macrophages and their precursors were affected. This syndrome has shown a genetic predisposition, with a greater frequency of occurrence in consanguineous offspring being noted in approximately one-third of the cases. It usually occurs during the first 4 to 5 years of age, and affects the primary dentition with a periodontal involvement, leading to an early exfoliation of the primary dentition. And as the permanent teeth erupt, the same sequence of events recurs, leading to the exfoliation of the permanent dentition. 46 47 This extreme condition is a huge challenge in total rehabilitation of the existing denture.

### Osteogenesis Imperfecta

Osteogenesis imperfecta is a syndrome characterized by low-bone mass and a marked increase in bone fragility. The disease is most often caused by mutations in the COL1A1 and COL1A2 genes, but recent work has shown that mutations in the CRTAP, LEPRE, and PPIB genes, which form a protein complex necessary for prolyl-3-hydroxylation of collagen, can cause recessive forms of osteogenesis imperfecta. 48 49

### Ectodermal Dysplasia

The term ectodermal dysplasia is used to designate a heterogenous group of disorders characterized by a constellation of findings involving a primary defect of the skin, teeth, and appendageal structures including hair, nail, exocrine and sebaceous glands. Dental manifestations include conical or pegged teeth, hypodontia or complete anodontia, and delayed eruption of permanent teeth. 50 The different types of ectodermal dysplasia are caused by the mutation or deletion of certain genes like EDA, EDAR, and EDARADD located on different chromosomes. 51

Hypohidrotic ectodermal dysplasia (HED) is usually transmitted as an X-linked recessive trait in which the gene is carried by the female and manifested in male. In X-linked form carrier mothers exhibit minimal expression in the form of hypodontia and/or conical teeth and spottily reduced sweating. 50 51

### Ehlers–Danlos Syndrome (EDS)

EDS is a genetic disorder that can be caused by mutations in several different genes, including COL5A1, COL5A2, COL1A1, COL3A1, TNXB, PLOD1, COL1A2, FKBP14, and ADAMTS2. Mutations in these genes usually change the structure, production, and/or processing of collagen or proteins that interact with collagen. Collagen provides structure and strength to connective tissues throughout the body. A defect in collagen can weaken connective tissues in the skin, bones, blood vessels, and organs, resulting in the signs and symptoms of EDS. 52 Early-onset generalized periodontitis is one of the most significant oral manifestations of the syndrome. This can lead to the premature loss of deciduous and permanent teeth.

### Chediak–Higashi Syndrome (CHS)

It is a disease of intracellular vacuolar and granule fusion caused by autosomal recessive mutations to the CHS1 gene (the human equivalent of the mouse LYST gene). CHS has both hematopoietic and neurological manifestations. Clinical symptoms include recurrent infections, with organisms normally eliminated by phagocytosis, peripheral neuropathy, partial oculocutaneous albinism, slight mental retardation, platelet dysfunction, severe periodontitis, and a sometimes fatal infiltration of lymphocytes and macrophages into various organs.

Periodontal disease and bone loss of dental alveoli associated with various microorganisms are common. The homeostasis of neutrophils is tightly regulated through coordinated bone marrow production, release into the circulation, transmigration to and activation in peripheral tissues, and clearance of senescent neutrophils. Dysregulation of any of these homeostatic mechanisms at any age can cause severe periodontitis. Accordingly, both impaired and excessive neutrophil activity (in terms of numbers or immune function) can precipitate periodontitis. Neutrophil defects of congenital origin (e.g., congenital neutropenia, leukocyte adhesion deficiency, and CHS) are associated with cutaneous and systemic infections and early-onset forms of periodontitis, affecting both the primary and permanent dentitions of children. 53 54

## Conclusion

Bone, dental loss, and defects caused by diseases have become a global concern, with high incidence, which seriously affects the health and life quality of the whole population. At many times, it is best to attempt to intervene in the disease , as it is challenging to remove the cause at the genetic level. Dentists could be the first persons to identify these rare conditions at an early stage, making it particularly necessary for them to adequately grasp the related clinical features. Understanding the effects of genes on bone modulation will shed more light on the pathogenesis and therapeutics of bone and teeth.
